# Draft Genome Sequence of the Alkaliphilic, Lithoautotrophic Homoacetogen Fuchsiella alkaliacetigena Z-7100^T^

**DOI:** 10.1128/mra.00471-22

**Published:** 2022-08-01

**Authors:** Yan Huang, Masaru K. Nobu, Kensuke Igarashi, Souichiro Kato

**Affiliations:** a Graduate School of Agriculture, Hokkaido University, Sapporo, Japan; b Bioproduction Research Institute, National Institute of Advanced Industrial Science and Technology, Sapporo, Japan; c Bioproduction Research Institute, National Institute of Advanced Industrial Science and Technology, Tsukuba, Japan; University of Delaware

## Abstract

Fuchsiella alkaliacetigena is a spore-forming, alkaliphilic hydrogentrophic homoacetogen that was isolated from the soda lake Lake Tanatar III in Russia. The genome of the type strain Z-7100 (= DSM 24880) is 2.9 Mb, with a G+C content of 36.2%.

## ANNOUNCEMENT

Fuchsiella alkaliacetigena Z-7100^T^ was an obligate and extreme natronophile belonging to the family *Halobacteroidaceae* of the order *Halanaerobiales* (1). It was an alkaliphilic acetogen that could grow lithotrophically with H_2_ plus CO_2_, which was similar to the family members Fuchsiella ferrireducens and Acetohalobium arabaticum but different from Natroniella acetigena (1–4). The strain Z-7100^T^ could utilize only limited substrates, i.e., ethanol, lactate, and pyruvate, with acetate and CO_2_ as the only metabolic products (1). Nitrate, selenate, anthraquinone-2,6-disulfonate (AQDS), and trace thiosulfate could be used as electron acceptors (1).

F. alkaliacetigena Z-7100^T^ was anaerobically cultivated at 40°C using DSMZ medium 1352. The genomic DNA was extracted from an exponentially growing culture using the MasterPure Gram-positive DNA purification kit (Epicentre) following the manufacturer’s instruction. The extracted DNA was used to construct Illumina shotgun paired-end sequencing libraries using the TruSeq DNA PCR-free library preparation kit (Illumina) and then was sequenced with the Illumina HiSeq 2000 platform (100-bp paired-end reads). A total of 22,931,582 read pairs (a total of 45,863,164 reads or 4.59 Gbp) were obtained. Quality trimming was performed using fastp v0.20.1 (–detect_adapter_for_pe –W 6 –M 20 –r –l 78) (5). *De novo* assembly with SPAdes v3.14.1 (–isolate –k 21, 33, 41, 65, 77) (6) generated 116 contigs (>1,000 bp) with a total of 2.9 Mb. The assembly was estimated to have a completeness of 99.14% by CheckM v1.0.18 (7) using KBase (8). The G+C content of the draft genome was 36.2%. Gene prediction with Prokka v1.14.0 (default parameters) (9) yielded 2,747 genes, comprising 2,700 protein-encoding genes and 46 RNA genes, including 3 rRNA genes, 43 tRNA genes, and 1 transfer-messenger RNA. The genome contains genes encoding the enzymes of the Wood-Ljungduhl pathway, which is responsible for carbon fixation. Genes encoding F-type ATPase, the electron transport complex Rnf, and several hydrogenases associated with energy conservation were also present. In addition, genes encoding enzymes for pyruvate oxidation to acetyl-coenzyme A (CoA) were present in the genome. The availability of this draft genome will aid in research on the metabolism mechanism and adaption strategies of haloalkaliphilic acetogens for the extreme environment.

### Data availability.

This draft genome and the raw sequence data have been deposited in NCBI GenBank and Sequence Read Archive (SRA) under the accession numbers JALKBZ000000000, and SRR19261504, respectively.
